# The time course of hypoxia effects using an aviation survival trainer

**DOI:** 10.3389/fcogn.2024.1375919

**Published:** 2024-04-10

**Authors:** Cammi K. Borden, Daniel G. McHail, Kara J. Blacker

**Affiliations:** ^1^Naval Medical Research Unit-Dayton, Brain & Cognitive Sciences Department, Wright-Patterson AFB, Dayton, OH, United States; ^2^Oak Ridge Institute of Science and Education (ORISE), Oak Ridge, TN, United States

**Keywords:** hypoxia, altitude simulation, aviation training, event-related potentials, human performance

## Abstract

**Introduction:**

Reduced environmental oxygen levels at high altitudes can result in hypoxic hypoxia, which remains a primary threat in tactical aviation. Hypoxia broadly impairs cognition and can degrade a pilot's ability to safely operate the aircraft. Current hypoxia countermeasures include aircraft life support systems that deliver supplemental oxygen and using controlled hypoxia exposures to train aviators to recognize symptoms. To maximize the effectiveness of these countermeasures, it is critical to understand how hypoxia impacts performance and associated neurocognitive outcomes. We previously showed that a neural marker that indexes sensory processing integrity is sensitive to hypoxia impairment.

**Methods:**

Here, we extend this line of research closer to the training environment by using hypoxia simulation equipment currently standard in aviation survival training. In a single-blind, repeated-measures, counterbalanced design, we exposed 34 healthy participants to either normoxic air (ground level) or normobaric hypoxia (altitude equivalent gradually increasing from 10 to 25k') for 20 min after a 10 min baseline at ground level. During the exposure, participants completed a cognitive assessment battery while passively elicited neural responses to auditory tones were recorded using electroencephalography (EEG). Participants reported their hypoxia symptoms throughout and upon completion of their exposures.

**Results:**

We found that the hypoxia exposure rapidly elicited the predicted physiological responses in peripheral oxygen saturation (decrease) and heart rate (increase) within 2–3 minutes of exposure onset. On average, participants reported hypoxia symptoms in a delayed manner, ~8 min following the exposure onset. Performance on the cognitive tasks was relatively unaffected by hypoxia for basic tasks including Stroop, fine motor tracking, color vision and arithmetic, but was significantly degraded by hypoxia for more advanced tasks that combined a visual search component with Stroop and a working memory task. EEG activity associated with pre-attentive auditory processing was impaired on average shortly after the first symptom report, ~10 min from exposure start.

**Discussion:**

Together, these results move hypoxia research closer to conditions encountered in aviation survival training and support the use of training devices for future hypoxia research.

## 1 Introduction

Hypoxic hypoxia results from reduced levels of breathable oxygen. Healthy individuals may encounter hypoxic conditions during mountain climbing or aviation at high altitudes. Hypoxia impairs many perceptual and cognitive processes (Fowler et al., 1987; Temme et al., 2010; Malle et al., 2013). In tactical aviation, hypoxia represents a risk to safety and performance and has been suggested as a possible cause of physiological episodes (PEs). The threat of hypoxia in tactical aircraft has recently gained increased recognition in the military aviation community (Elliott and Schmitt, 2019). Current countermeasures for hypoxia in military aviation include training aviators to recognize symptoms of hypoxia and execute emergency procedures during a controlled exposure to hypoxia, such as in the Naval Aviation Survival Training Program (NASTP). However, self-recognition of hypoxia symptoms may be delayed or inconsistent (Cox et al., 2024). More robust psychological or physiological indicators are necessary to supplement training and in-cockpit systems that monitor hypoxia-related impairment.

Acute hypoxia exposure has been shown to negatively impact performance on several functions that are relevant to aviators. Firstly, hypoxic environments have been shown to increase response time (RT) latency (Fowler et al., 1982, 1987; Fowler and Lindeis, 1992; Shukitt-Hale et al., 1998; Dart et al., 2017). The visual system is profoundly impacted by hypoxia, ranging from impaired color vision (Connolly et al., 2008; Barbur and Connolly, 2011), perceived light intensity (Fowler et al., 1993), and other commonly reported symptoms such as graying, tunnel vision, and blurry vision (Woodrow et al., 2011). Higher-order cognitive functions have also shown sensitivity to hypoxia, such as working memory (Shukitt-Hale et al., 1994; Malle et al., 2013; for a review see, McMorris et al., 2017) and decision making (Legg et al., 2012; Niedermeier et al., 2017). For example, at 31 k' in a hypobaric chamber, participants were significantly worse at a classic measure of working memory (i.e., Paced Auditory Serial Addition Task) and there was a positive correlation between peripheral oxygen saturation (SpO_2_) and task accuracy (Malle et al., 2013). Moreover, complex reasoning tasks that involved conflict decisions were particularly impacted by hypoxia in one prior study comparing 8 k' and normoxia (Legg et al., 2012). These effects of hypoxic exposure for aviators may translate to an impaired ability to maintain airspeed, altitude, and directional heading (Green and Morgan, 1985; Cable, 2003; Temme et al., 2010; Steinman et al., 2017).

This rich literature on acute hypoxia and performance highlights that a number of important sensory and cognitive functions are impaired under these conditions. However, assessing cognitive function with behavioral measures as an indicator of hypoxia during aviation is unrealistic. While cognitive impairment is the end state that we want to prevent with physiological monitoring, the aviation community needs a quick and non-invasive approach to detecting impairment. Recent studies using electroencephalography (EEG) during hypoxic exposures have uncovered neural markers associated with sensory and cognitive deficits due to hypoxia impairment (Seech et al., 2020; Blacker and McHail, 2021, 2022; Blacker et al., 2021). EEG is a non-invasive measure of brain activity, which allows for assessment of sensory and cognitive processing and produces quantifiable electrophysiological signatures called event-related potentials (ERP). One passive ERP paradigm that reflects the attentional re-orienting after the presentation of a novel stimulus has been shown to be disrupted during acute hypoxia (Seech et al., 2020; Blacker and McHail, 2021; Blacker et al., 2021). The ERP, known as the mismatch negativity (MMN) and P3a complex, occurs in response to infrequent auditory stimuli that are interspersed with and differ slightly from more frequent auditory stimuli (called the “oddball” paradigm). Importantly, this ERP is pre-attentive, as the tones are played in the background while participants are engaged with a different task. Previous studies using an auditory oddball paradigm found a significant reduction in the amplitude of the P3a component during hypoxic exposure compared to a normoxic exposure (Seech et al., 2020) and reduced amplitude and delayed recovery of the MMN component (Blacker and McHail, 2021). Another study using visual stimuli also demonstrated a reduced amplitude in the visual MMN component, which reflects the initial orienting of attention to the deviant stimulus (Blacker et al., 2021). These studies demonstrate that passively elicited ERPs can track deficits in early sensory information processing during an acute hypoxia exposure.

The MMN/P3a complex was originally chosen to study with acute hypoxia because it is a well-studied neural signature that can reliably index automatic and preattentive stages of early auditory information processing (e.g., Naatanen et al., 1978, 2007, 2019). Specifically, this neural response can be found in rodents, non-human primates, fetuses, sleeping infants and adults, and even comatose individuals (Javitt et al., 1994; Amann et al., 2010; Gil-Da-Costa et al., 2013; Swerdlow et al., 2013; Todd et al., 2013; Featherstone et al., 2015). Relevant for the study of acute hypoxia, this ERP complex is known to be sensitive to other central nervous system perturbations including pharmacological interventions and cognitive training (e.g., Dulude et al., 2010; Perez et al., 2017) and therefore has been targeted as a potential biomarker for central nervous system dysfunction (Light and Braff, 2005; Light and Naatanen, 2013). Despite the robust and automatic nature of the oddball paradigm to elicit the MMN/P3a complex, these neural processes do require energy that is linked to the oxygen supply in the brain. Therefore, decrements in breathable oxygen are likely to disrupt even early sensory information processing. Indeed, there is much prior work demonstrating that the electrical activity of the brain, as measured with EEG, is sensitive to its oxygen supply.

In addition to prior work using EEG/ERPs in the auditory domain, there is also evidence that visuo-spatial processing and sustained attention changes under hypoxic conditions can be indexed by EEG measures. For example, Altbacker et al. (2019) assessed the sensitivity of three variants of the P300 component (i.e., Target P3, No Go P3, and Novelty P3) to hypoxia evoked in response to a modified continuous performance task. The results demonstrated that the Novelty P3 amplitude, but not the other components, was significantly decreased under hypoxic compared to normoxic conditions. The authors concluded that novelty detection was impaired while task-relevant information processing and inhibitory functions were unaffected. Additionally, there is evidence that alpha power desynchronization may underlie attentional orienting changes that occur during acute hypoxia (Zani et al., 2020). Together, there is a robust literature on both the behavioral and neural consequences of acute hypoxia, which suggests that EEG may indeed be a strong candidate for further development as an indicator of hypoxia related impairment.

The previous studies used a mask-off exposure via a Reduced Oxygen Breathing Environment (ROBE), a normobaric chamber, for the hypoxia exposure. However, the NASTP recently adopted the On-Demand Hypoxia Trainer (ODHT; Lynntech Inc., 2020) and its successor, the Flight Breathing Awareness Trainer (FBAT), for hypoxia training, which more closely simulates breathing conditions in tactical aircraft life support systems. In the current study, we advance previous hypoxia research closer to the training environment by assessing changes in physiology, cognitive performance, and neural activity (MMN/P3a) during exposure to mask-on hypoxia using the ODHT.

## 2 Materials and methods

### 2.1 Participants

A total of 34 healthy adults (age: *M* = 29, *SD* = 5.7; 22 males, 12 females) participated for monetary compensation. All participants were recruited through flyers and online announcements. Participants who completed the study received $125. The study protocol was approved by the Naval Medical Research Unit – Dayton's (NAMRU-D) Institutional Review Board in compliance with all applicable federal regulations governing the protection of human participants. All participants self-reported normal or corrected-to-normal vision; normal hearing; no history of psychological, neurological, or medical diagnosis; no use of tobacco in the past 6 months; and no excessive alcohol use.

### 2.2 Tasks and procedures

#### 2.2.1 Hypoxia awareness tool (HAT)

**Figure 1:**
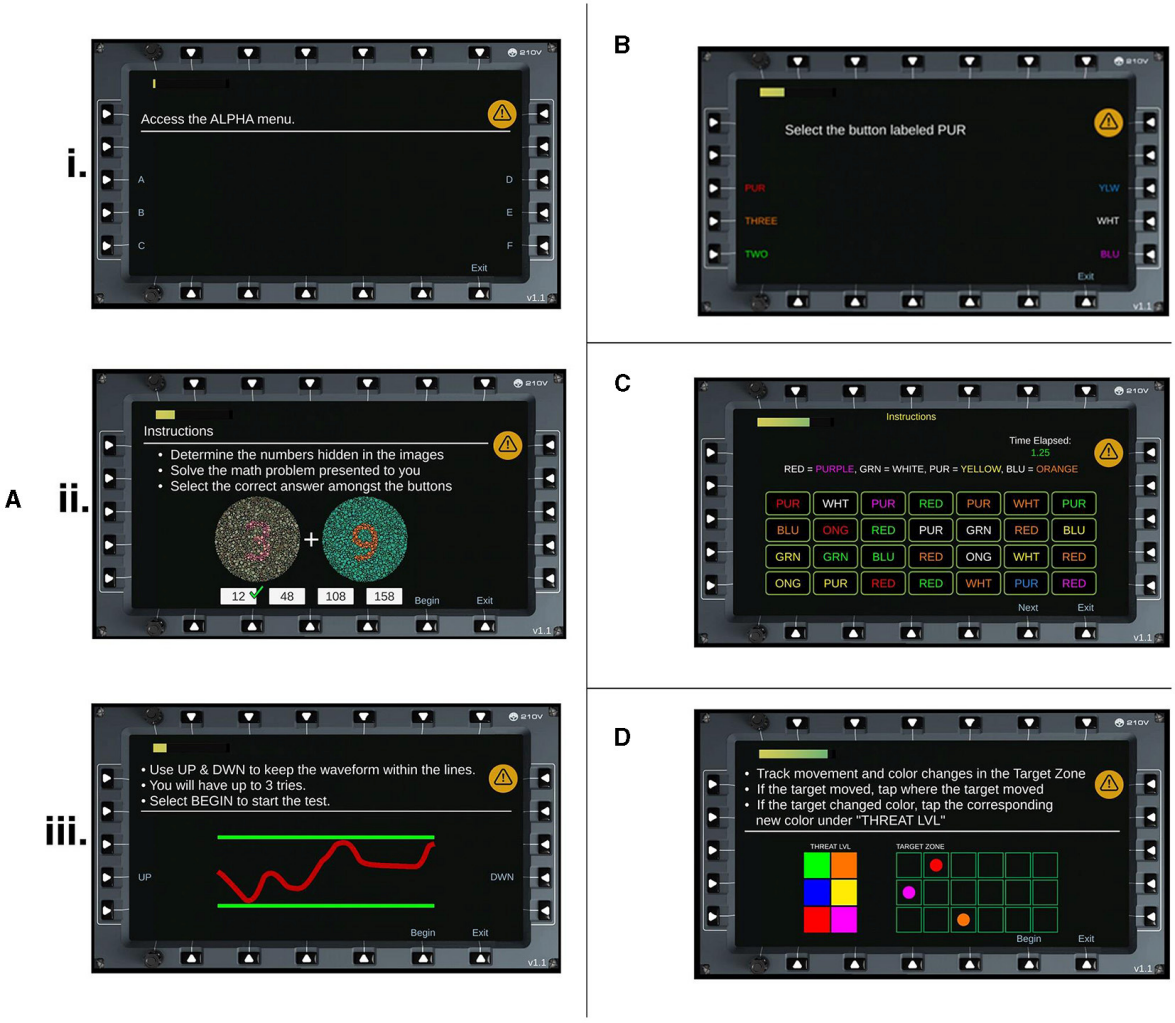
HAT Levels. Screenshots of each task and subtask for the HAT. **(A)** Level 1, Basic Tasking is divided into three subtasks (i) Rule operations (ii) Ishihara addition problems and (iii) Fine motor control. **(B)** Level 2, Stroop Task. **(C)** Level 3, Advanced Stroop. **(D)** Level 4, Tracking Task.

Participants were seated ~60 cm from a 13.5” tablet. The HAT program (SoarTech Inc.) consisted of a series of cognitive tasks that required following instructions and performing operations that are relevant to aircrew. The tasks were programmed in Unity and are available upon request from the corresponding author. The program was divided into four discrete levels. Level 1, referred to as Basic Tasking, consisted of three different subtasks. The first subtask required a series of rule operations to be followed by tapping on different menu options (e.g., “Access the Alpha menu” meant press the button next to the letter A; *n* = 10 trials). Each trial increased in complexity of the operations to follow. The second subtask required participants to complete simple addition problems, with the numbers to sum displayed in Ishihara color testing displays (*n* = 4 trials). Participants chose the answer from four alternative choices presented. Finally, Level 1 ended with a fine motor task that required constant adjustment to keep a trace in between two parallel boundary lines (*n* = 1 trial). Participants were given three attempts in the event they failed. Level 2, referred to as Stroop, required participants to find and select the appropriate stimulus among six alternatives (e.g., select button labeled RED, written in all white; *n* = 5 trials). Level 3, referred to as Advanced Stroop, stated four Level 2 stimuli to be found and selected amongst an array of 28 possibilities (i.e., a 4 × 7 grid of choices). For example, RED = GREEN meant find the word RED in green ink. The task was timed to increase difficulty (*n* = 3 trials). Finally, Level 4, referred to as Target Tracking, was a multiple-object tracking task that required participants to track the color and spatial location of seven circle stimuli in a 3 × 6 grid (*n* = 20 trials). Six color options were used and 18 possible locations. Any change to location or color of a stimulus required a response. Screenshots of each level can be seen in Figure 1. The duration of these tasks varied between participants, though each level could be completed in 2 min or less. Participants were instructed to focus primarily on these tasks and answer as accurately and as quickly as possible.

#### 2.2.2 EEG data acquisition and oddball paradigm

EEG data were recorded continuously from 32 electrodes covering the whole scalp with approximately uniform density using an elastic electrode cap (ActiCHamp, Brain Products) referenced to the right mastoid (TP9) in DC mode, at a sampling rate of 1000Hz. A standard 10–20 montage was used. Electrode impedance for all channels was kept below 10 kΩ.

Auditory stimuli were presented for the same 30 min period as the HAT was performed. Participants were instructed to ignore the tones and focus on the HAT. The auditory tones were presented to participants every 500 ms and at 85 dB sound pressure level via Etymotic ER3-A insert earphones. The passive auditory oddball paradigm comprised a sequence of tones, of which, 85% were standards (50 ms, 1000 Hz, *n* = 2,805) and 15% were deviants (*n* = 495 per deviant type), where the tones either differed in duration (100 ms), frequency (1100 Hz), or both (100 ms, 1100 Hz). These different deviant types were selected based on previous work demonstrating that these conditions have excellent test-retest reliability, strong associations with cognition and daily functioning, and sensitivity to interventions (Swerdlow et al., 2016, 2018; Perez et al., 2017, 2019; Hochberger et al., 2019; Light and Swerdlow, 2020). All tones had 5 ms rise/fall.

#### 2.2.3 Symptom reporting

For both visits, participants reported subjective symptoms using two different methods. The first method was in real-time during the exposure, where participants were given a list of common hypoxia symptoms (Sausen et al., 2001) and were instructed to point to a symptom when they recognized the onset of it during their exposure. Participants familiarized themselves with the list prior to the start of the exposure. An experimenter observed the participant during the exposure, and promptly recorded the indicated symptom and the timestamp. Participants were instructed to avoid reporting symptoms that had other causes (e.g., headache from the headgear) and to try to only report symptoms they thought were caused by potential hypoxia. The second method of reporting was via the Hypoxia Symptom Questionnaire (HSQ; Sausen et al., 2001) administered after the exposure. The HSQ asks participants to report symptom severity on a 4-point scale (0 = not observed, 1 = mild, 2 = moderate, and 3 = severe). The lists administered to the participants between the real-time and HSQ varied slightly. The HSQ did not include several symptoms that were listed for the real-time (e.g., sweating, lightheaded, heart rate, numbness, and difficulty concentrating). The HSQ also listed three specific visual changes (e.g., light dimming, tunnel vision, and blurred vision), whereas the real-time listed only “visual changes.”

#### 2.2.4 Breathing exposure

Gas was delivered to the participant via the ODHT connected to a 20-P flight mask worn by the participant. The ODHT is an Electrochemical Oxygen Separation device that provides pressure-on-demand flow rate designed to simulate the air delivery method used in aircraft life support systems. The ODHT delivers breathing gas to the participant and induces a normobaric hypoxia exposure by adjusting the nitrogen/oxygen content in the gas mixture. Two profiles were created via the ODHT for the normoxia and hypoxia exposures, which are detailed below.

#### 2.2.5 Physiological monitoring

During delivery of gas mixtures via the ODHT, both peripheral oxygen saturation (SpO_2_) and heart rate (HR) were monitored and recorded at a sampling rate of 1Hz. Both measures were acquired via a Nonin finger-mounted pulse oximeter (Nonin Medical Inc., 2015) and recorded by an iPad via Bluetooth connection. A safety cut-off criterion of 55% SpO_2_ was used.

#### 2.2.6 Experimental procedures

**Figure 2:**
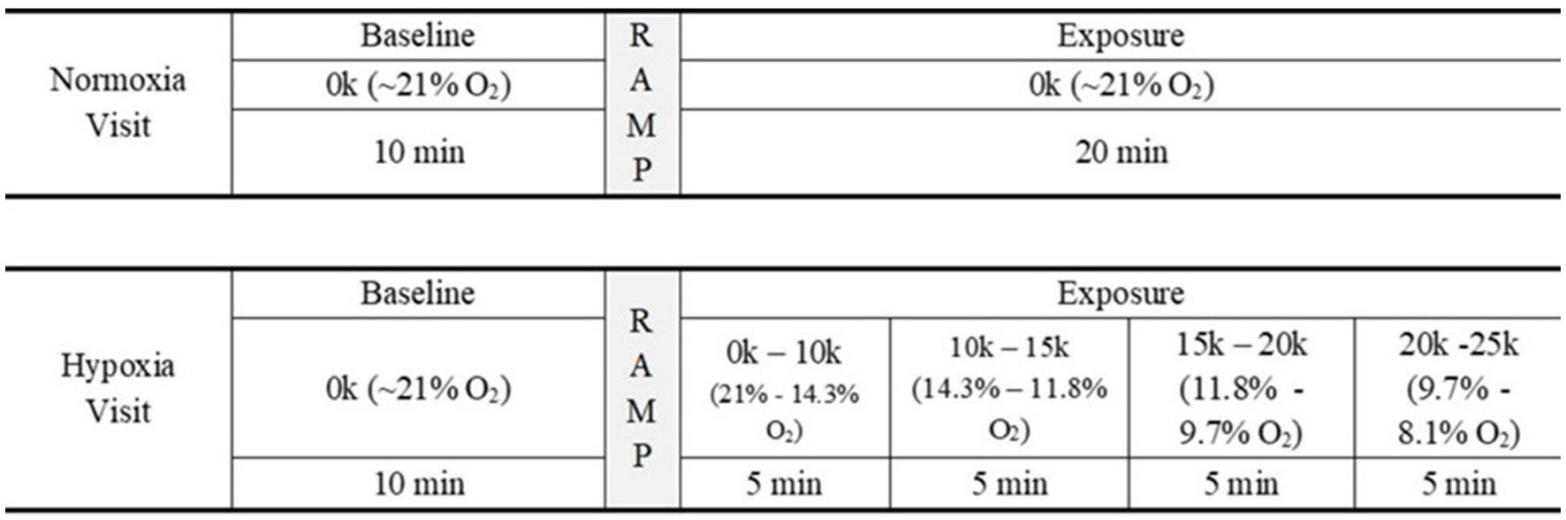
Study design. Schematic of the study's experimental design. For 30 min, participants attended to the HAT and passively listened to auditory stimuli while EEG data were recorded.

Participants completed two visits on separate days. Figure 2 shows an overview of each study visit and the relevant activities in each visit. On the first visit, participants were instructed on how to perform the HAT and completed at least one full round of the HAT prior to being connected to the ODHT. In addition, participants were instructed on symptom reporting and became familiarized with the symptom list prior to being connected to the ODHT. Participants then performed consecutive rounds of the HAT while passively listening to auditory stimuli and having EEG recorded for up to 30 min of testing.

Each visit consisted of up to 30 min of data collection. Both visits began with a 10 min baseline period, where the participant breathed normal 21% oxygen via the ODHT. For the normoxia visit, the 20 min following the baseline period (i.e., the “exposure” period) involved continuing to breathe 21% oxygen. For the hypoxia visit, after the 10 min baseline period, the ODHT began to ramp up in simulated altitude and followed a set schedule: 5 min at 10k ft (14.3% oxygen), 5 min at 15k ft (11.8% oxygen), 5 min at 20k ft (9.7% oxygen), and 5 min at 25k ft (8.1% oxygen). Participants were blind to the condition, and the order of testing days was counterbalanced across participants. Following the exposure, participants were administered a recovery gas of 35% oxygen, were disconnected from the ODHT and EEG, completed the HSQ, and then were dismissed.

#### 2.2.7 EEG data analysis

EEG data were processed using the Fieldtrip software package (Oostenveld et al., 2011). Data were segmented into epochs covering the time from 100 ms before to 500 ms after the onset of each auditory stimulus presentation. After trial epochs were created, data were low-pass filtered at 20 Hz (Luck, 2014), and re-referenced to the average of both mastoids. Independent components analysis (ICA) was performed on epoched data, and the eye blink and lateral eye movement components were removed for every participant. After ICA, EEG waveforms from frontal electrodes (i.e., Fp1, Fp2) were visually inspected to identify voltage fluctuations typical of artifacts (amplitude > ±75 μV). Trials containing artifacts were rejected entirely.

After artifact rejection, average waveforms were calculated for deviant and standard stimuli responses within 6 min windows throughout each visit, using electrode Fz. A window length of 6 min was chosen based on an analysis of normoxia data that showed reliable MMN/P3a mean amplitudes at ~6 min (see Supplementary Figure 1). Data for each visit was aligned by exposure start time, such that the beginning of each adjusted visit was 9 min prior to the exposure start. To assess how responses varied over time, waveforms were analyzed within 6 min windows beginning with the start of the visit and advancing in 20 s increments, such that the second window occurred from 0:20 to 6:20, and so on through the remainder of the visit. Difference waves were calculated for each 6 min window by subtracting the averaged standard response from the averaged deviant response in each window. As in Blacker et al. (2021), MMN and P3a amplitude were determined by finding the average difference wave amplitude in a 50 ms window centered around the local minimum/maximum for the ranges 150–250 ms and 200–350 ms post-stimulus, respectively. MMN/P3a mean amplitude and peak-to-peak amplitude were found separately for each 6-min window in each visit to analyze changes in these characteristics throughout the visit and varying exposure conditions.

### 2.3 Statistical analysis

The current study was a within-subjects design whereby participants completed the normoxia and hypoxia conditions on separate days in counterbalanced order. Participants were blinded to which condition was presented on each visit. The primary statistical analysis approach was to examine the two conditions (hypoxia, normoxia) and time (baseline, exposure).

The different levels of the HAT were self-paced, thus the time to complete one round of the HAT varied across participants. Therefore, the performance of the second and third rounds (i.e., first sets) as well as the last two full rounds (i.e., last sets) for each participant were averaged and selected for analysis. The first round was deemed a practice round and excluded from analysis.

Two approaches were used to analyze the ERP data. First, to examine the time at which our ERP measures during the hypoxia exposure deviated from the normoxia exposure, we used a SPM paired *t*-test (Pataky, 2010). In the second method, we tested a repeated-measures ANOVA to compare the baseline period to the last 10 min of the exposure period. Given the paired-samples nature of these tests, we included all participants that completed a minimum of 25 min of the hypoxia visit (*n* = 27).

## 3 Results

Of the 34 participants who enrolled in the study, three participants did not complete both visits. Therefore, 31 participants were retained for subsequent analyses. In addition, the Greenhouse-Geisser correction was applied where Mauchly's test showed that the sphericity assumption was violated.

### 3.1 Cognitive performance

**Figure 3:**
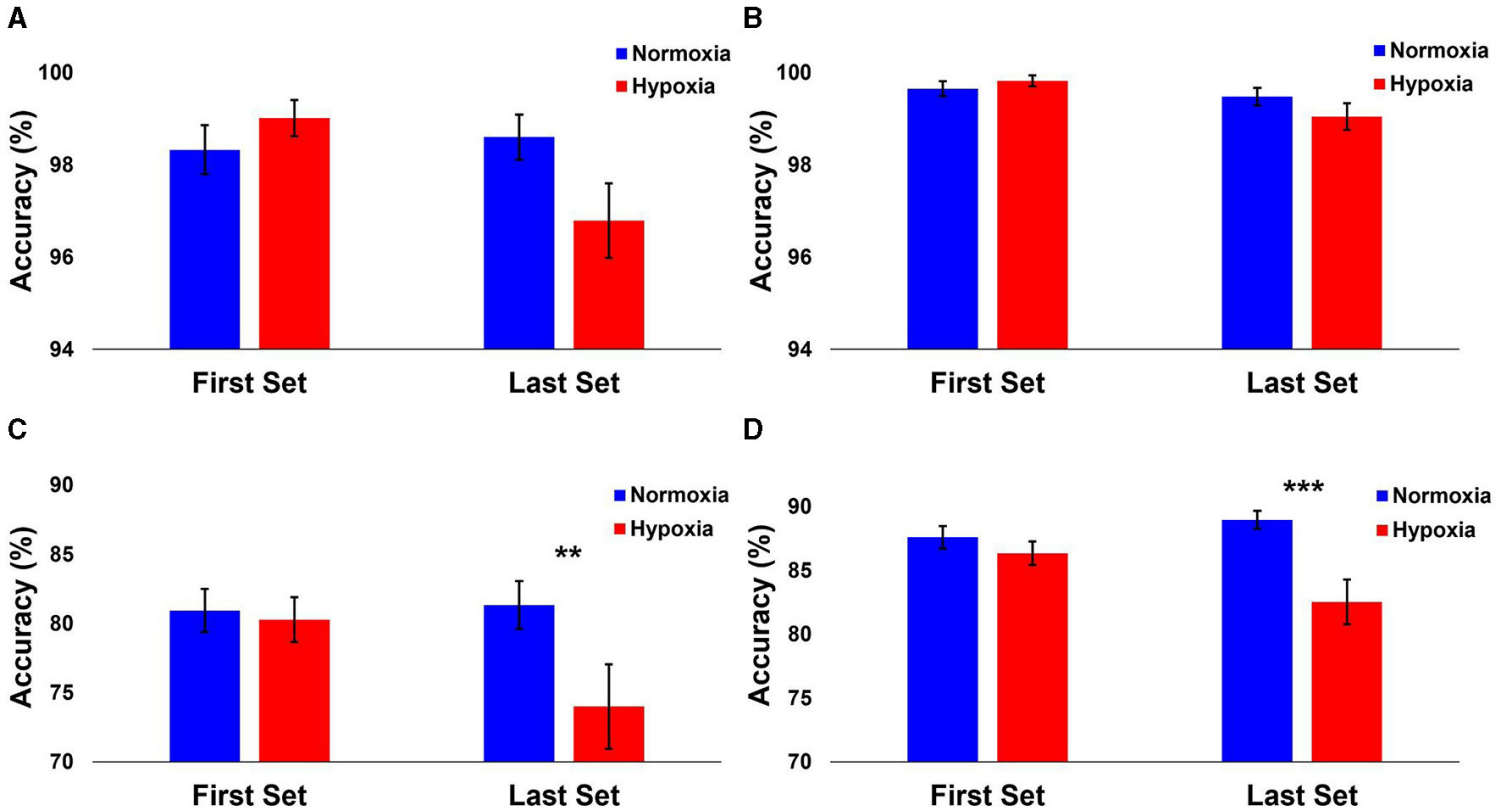
Performance results from each HAT level. The mean accuracy of each level shown separately for normoxia and hypoxia compared between the first and last sets. **(A)** Level 1: Operational Subtasks. There was a marginal significance from the last 2 rounds compared to normoxia and hypoxia (*p* = 0.058). **(B)** Level 2: Stroop Task. **(C)** Level 3: Advanced Stroop. A significant decrease between normoxia and hypoxia in the last 2 rounds (*p* = 0.005). **(D)** Level 4: Tracking Task. A significant decrease between normoxia and hypoxia in the last 2 rounds (*p* = 0.001). Error bars represent SEM. **p ≤ 0.01. ***p ≤ 0.001.

The following analyses include data from 29 participants, as two participants ended their hypoxia visit early and did not have enough data for the HAT. A 2 (condition: normoxia vs. hypoxia) × 2 (time: first sets vs. last sets) repeated-measures ANOVA was tested on the accuracy for each level separately. For Level 1, the main effect of condition [*F*_(1, 28)_ = 1.149, *p* = 0.293] and the main effect of time [*F*_(1, 28)_ = 3.364, *p* = 0.077] did not reach significance (Figure 3A). However, the condition × time interaction [*F*_(1, 28)_ = 4.177, *p* = 0.05, = 0.13] was significant. Planned paired samples *t*-tests showed a marginally significant decrease in accuracy on the last sets during hypoxia compared to normoxia [*t*_(28)_ = 1.979, *p* = 0.058, *d* = 0.37], but no difference between conditions on the first sets [*t*_(28)_ = −1.019, *p* = 0.317]. For Level 2, the main effect of time was significant [*F*_(1, 28)_ = 10.79, *p* = 0.003, $\eta_{p}^{2}$ = 0.278] with decreased accuracy on the last sets compared to the first sets (Figure 3B). However, the main effect of condition [*F*_(1, 28)_ = 0.383, *p* = 0.541] and the condition × time interaction [*F*_(1, 28)_ = 1.869, *p* = 0.182] did not reach significance. For Level 3, the main effect of time was significant [*F*_(1, 28)_ = 5.34, *p* = 0.028, $\eta_{p}^{2}$ = 0.16], again with decreased accuracy on the last sets compared to the first sets. The main effect of condition was significant [*F*_(1, 28)_ = 4.799, *p* = 0.037], with decreased accuracy during hypoxia compared to normoxia. Critically, the condition × time interaction was significant [*F*_(1, 28)_ = 10.413, *p* = 0.003, $\eta_{p}^{2}$ = 0.271]. Planned paired samples t-tests showed a significant decrease in accuracy on the last sets during hypoxia compared to normoxia [*t*_(28)_ = 3.065, *p* = 0.005, *d* = 0.57], but no difference between conditions on the first sets [*t*_(28)_ = 0.374, *p* = 0.711]. As shown in Figure 3C, participant performance was significantly impaired on the last sets during the hypoxia exposure for the Advanced Stroop. Finally, for Level 4, the main effect of condition was significant [*F*_(1, 28)_ = 9.627, *p* = 0.004, $\eta_{p}^{2}$ = 0.256], with worse performance during the hypoxia condition compared to normoxia. The main effect of time was not significant [*F*_(1, 28)_ = 2.04, *p* = 0.164], but critically, the condition × time interaction was significant [*F*_(1, 28)_ = 8.865, *p* = 0.006, $\eta_{p}^{2}$ = 0.24]. Planned paired samples *t*-tests revealed a significant decrease in accuracy on the last sets during hypoxia compared to normoxia [*t*_(28)_ = 3.564, *p* = 0.001, *d* = 0.66], but no difference between conditions on the first sets [*t*_(28)_ = 1.095, *p* = 0.283]. As shown in Figure 3D, participant performance was significantly impaired on the last sets during the hypoxia exposure for the Tracking Task.

### 3.2 ERP results

#### 3.2.1 MMN and P3a characteristics over time

**Figure 4:**
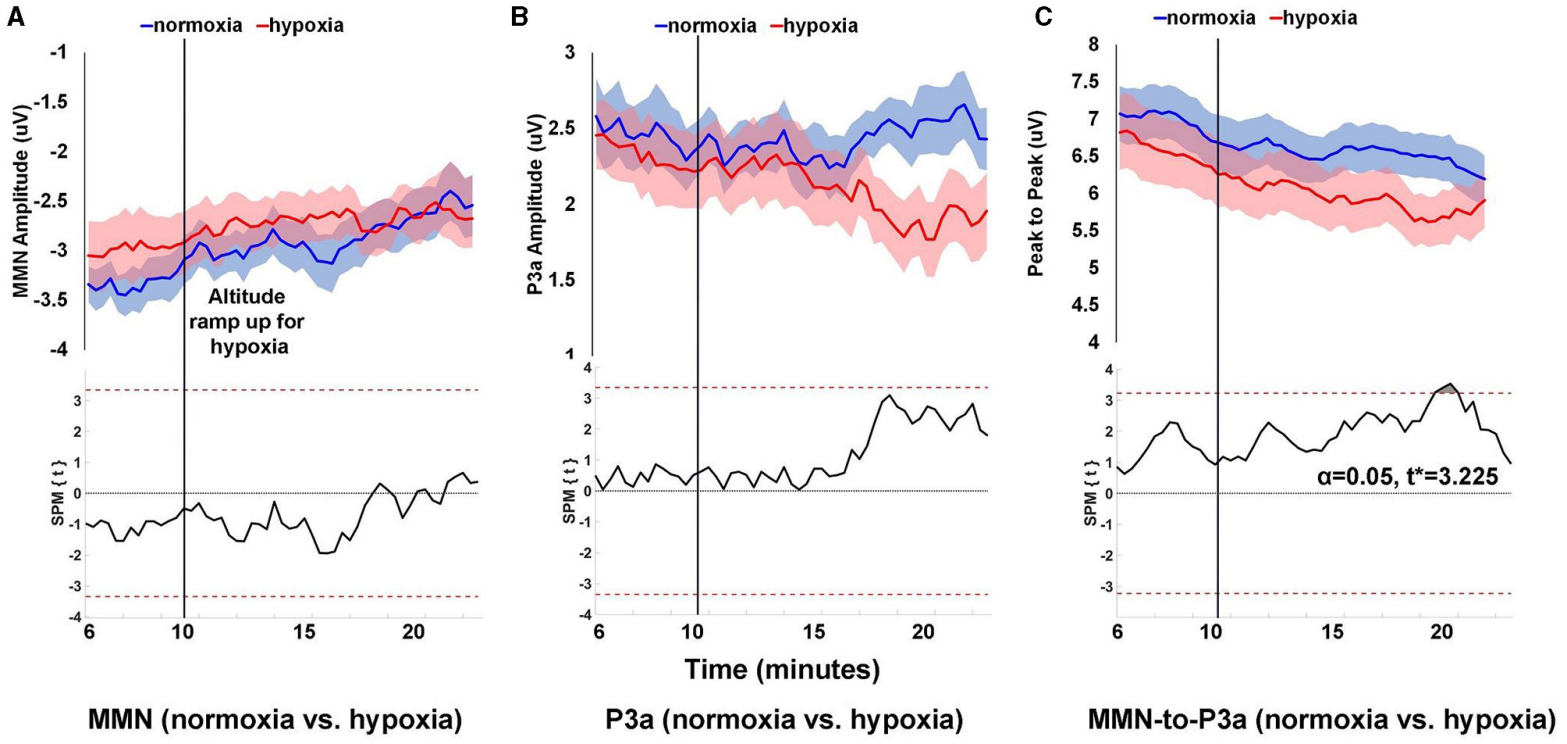
Peak amplitude over time for MMN **(A)**, P3a **(B)**, and peak-to-peak **(C)** components. Peak amplitude was calculated for each component within 6 min windows that began at the start of the visit and advanced in 20 s increments throughout the remainder of the visit. Shaded areas (upper panels) are SEM. The lower panels show SPM paired *t*-test results comparing amplitudes for normoxia and hypoxia visits, where the dashed red line is the threshold for statistical significance at α = 0.05.

Analysis of normoxia data suggested that 6 min of data were sufficient to generate a reliable MMN/P3a (see Supplementary Figure 1). Therefore, to assess how the MMN/P3a changed over time, average difference waves were calculated for each 6 min window of data beginning with 9 min prior to the start of the exposure period and proceeding every 20 s thereafter. MMN and P3a mean amplitude centered around the peak, and peak-to-peak amplitude were calculated for each window as shown in Figure 4.

For MMN amplitude, as shown in Figure 4A, the difference between normoxia and hypoxia never reached statistical significance with an α = 0.05 and critical t^*^ = 3.345. For P3a amplitude, as shown in Figure 4B, the difference between normoxia and hypoxia also never reached statistical significance with an α = 0.05 and critical t^*^ = 3.348. For peak-to-peak amplitude between the MMN and P3a, as shown in Figure 4C, 10 min into the exposure period, there was a significant reduction in the amplitude during the hypoxia compared to the normoxia visit, t^*^ > 3.225, *p* = 0.0291.

#### 3.2.2 MMN and P3a characteristics: baseline vs. last 10 min of exposure

**Figure 5:**
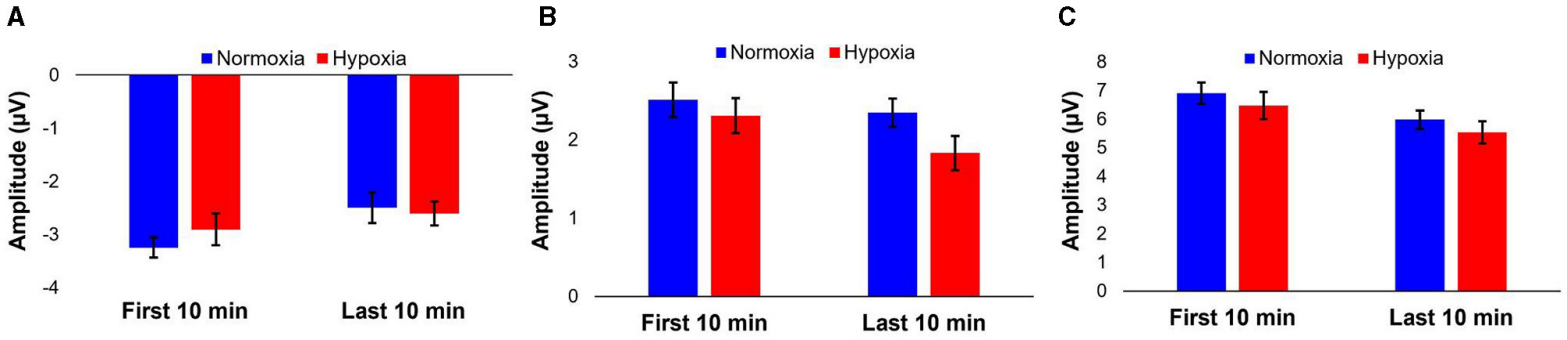
Average peak amplitudes of MMN **(A)**, P3a **(B)**, and peak-to-peak **(C)** components for the first 10 and last 10 min of the visit per condition. Error bars represent SEM.

A 2 (condition: normoxia vs. hypoxia) × 2 (time: baseline vs. last 10 min of exposure) repeated-measures ANOVA was tested for MMN and P3a amplitudes, as well as peak-to-peak amplitude. For the MMN amplitude, the main effect of time was significant [*F*_(1, 26)_ = 11.644, *p* = 0.002, $\eta_{p}^{2}$ = 0.309] with decreased amplitude for the last 10 min of the exposure compared to baseline, likely caused by habituation. The main effect of condition [*F*_(1, 26)_ = 0.434, *p* = 0.516] and the condition × time interaction [*F*_(1, 26)_ = 1.172, *p* = 0.289] were not significant. For the P3a amplitude, the main effect of time [*F*_(1, 26)_ = 4.743, *p* = 0.039, $\eta_{p}^{2}$ = 0.154], was significant with decreased amplitude for the last 10 min of the exposure compared to baseline, again likely caused by habituation. The main effect of condition was marginally significant [*F*_(1, 26)_ = 3.92, *p* = 0.058, $\eta_{p}^{2}$ = 0.131], with decrease amplitude during the hypoxia compared to the normoxia condition. The condition × time interaction [*F*_(1, 26)_ = 1.06, *p* = 0.313] did not reach significance. For the peak-to-peak amplitude of MMN to P3a, the main effect of time was significant [*F*_(1, 26)_ = 20.272, *p* < 0.001, $\eta_{p}^{2}$ = 0.438] with decreased amplitude for the last 10 min of the exposure compared to baseline, again likely caused by habituation. The main effect of condition was not significant [*F*_(1, 26)_ = 3.132, *p* = 0.089] nor was the condition × time interaction [*F*_(1, 26)_ = 0.00, *p* = 0.998]. Figure 5 illustrates these results.

### 3.3 SpO_2_, HR, and ODHT

**Figure 6:**
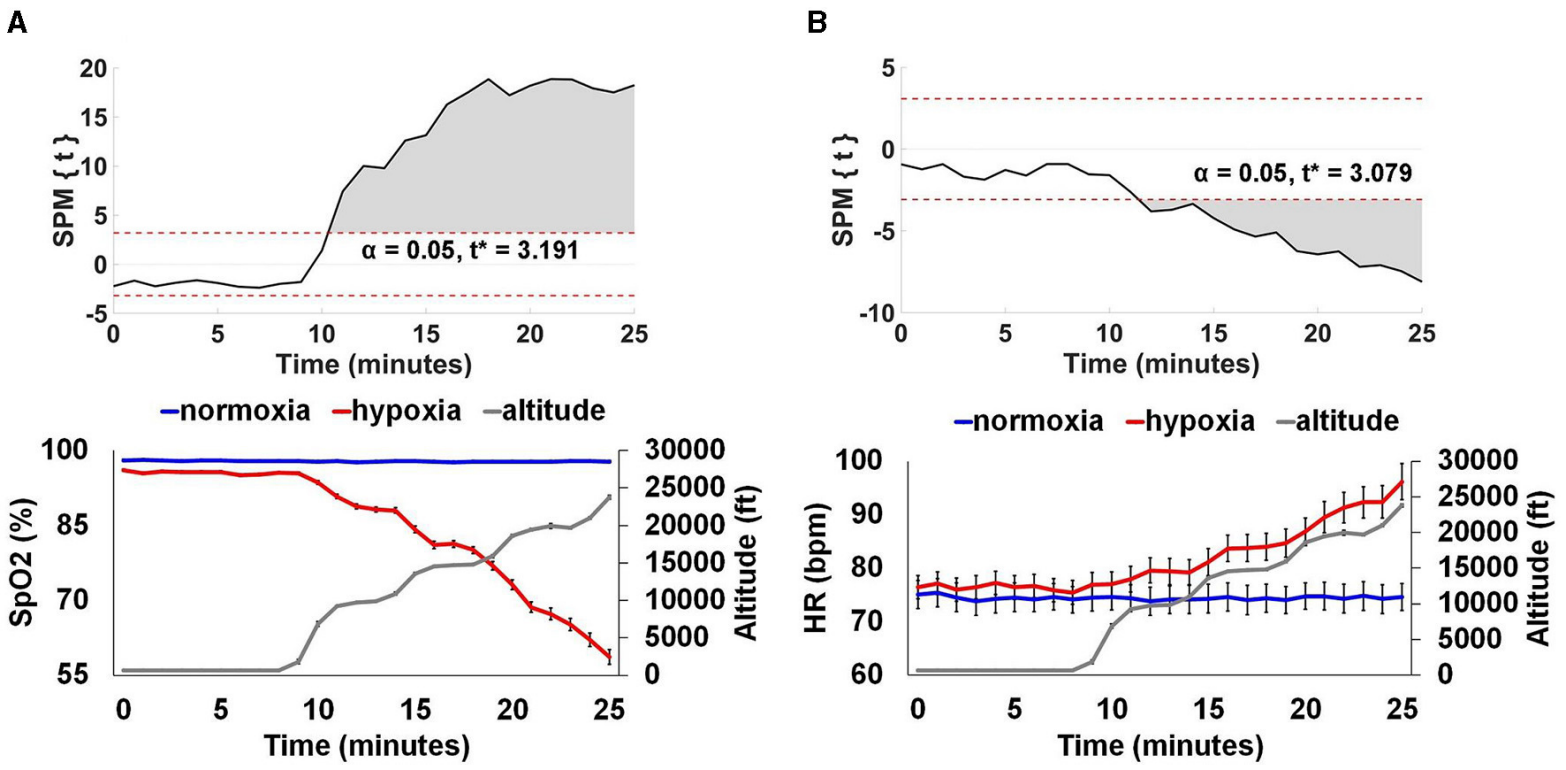
Physiological monitoring data. SpO_2_ **(A)** and HR **(B)**. The top panels show SPM paired t-test results showing where SpO_2_ and HR significantly diverged from normoxia for the hypoxia exposure. The bottom panels show group average (+/– SEM) SpO_2_ and HR for the normoxia and hypoxia visits separately, as well as the group average altitude presented by the ODHT. SpO_2_ was significantly reduced at 2 min, an average altitude of 9,239 ft and HR was significantly elevated at 3 min, an average altitude of 9,707 ft, *p* < 0.0001.

SpO_2_ and HR data were aligned by exposure start time and binned into 1 min averages across the duration of the two visits. To examine the time at which SpO_2_ and HR during the hypoxia exposure period deviated from the normoxia exposure, we used a statistical parametric mapping (SPM) paired *t*-test (Pataky, 2010). Given the paired-samples nature of this test, we included all participants that completed a minimum of 25 min of the hypoxia visit (*n* = 26). For SpO_2_, as shown in Figure 6A, at 2 min into the exposure period, there was a significant reduction in SpO_2_ during hypoxia compared to normoxia, t^*^ > 3.191, *p* < 0.0001. For HR, as shown in Figure 6B, at 3 min into the exposure period, there was a significant increase in HR during hypoxia compared to normoxia, t^*^ > 3.079, *p* < 0.0001. Figure 6 also shows the average profile of breathing gas throughout the 30 min visit for all participants that was delivered by the ODHT.

### 3.4 Hypoxia symptoms

The below data includes 28 participants, as one participant was excluded due to device error during the visit, and two noted at the end of their exposure periods that they forgot to report their symptoms during real-time report.

**Figure 7:**
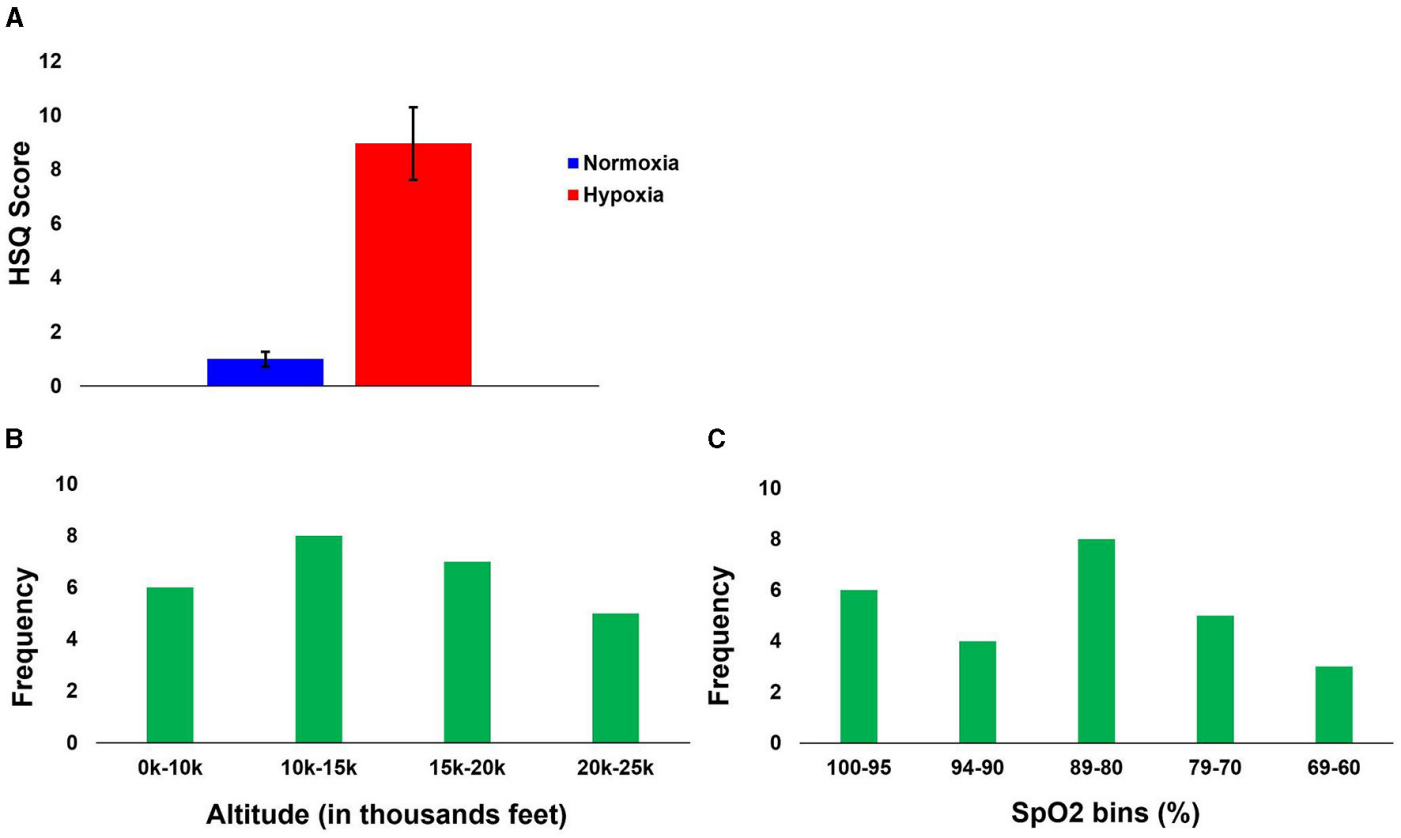
Hypoxia Symptom results. **(A)** Group average Total HSQ scores for both conditions. **(B)** First symptom reported relative to altitude. **(C)** First symptom reported relative to SpO_2_.

Per our previous study (Blacker and McHail, 2022), we tested a repeated-measures ANOVA on HSQ total score with condition (hypoxia vs. normoxia) as a factor. The main effect of condition was significant [*F*_(1, 27)_ = 33.232, *p* < 0.001, $\eta_{p}^{2}$ = 0.552], with increased symptom reporting during hypoxia compared to normoxia. Figure 7A shows the HSQ total score averages. In addition, Table 1 shows the frequency with which each HSQ item was reported (with any severity) for both conditions.

**Table 1:** Frequency of symptoms reported on the HSQ for both conditions, regardless of severity reported.

**Symptom**	**Normoxia**	**Hypoxia**
Tingling	2	19
Dizziness	1	17
Loss of coordination	3	16
Tunnel vision	3	15
Apprehension	0	14
Fatigue	4	13
Headache	4	13
Blurred vision	2	13
Breathlessness	1	13
Hot flashes	0	11
Light dimming	1	6
Nausea	1	6
Cold flashes	0	6
Euphoria	2	2
Loss of consciousness	0	2

*n* = 28.

During real-time reporting, participants reported their first symptom during hypoxia at an average of 8.03 min (*SD* = 6.96 min) into the exposure period, with a minimum time to report of 27 s and a maximum time to report of 19.25 min after the exposure onset. During the normoxia visit, a total of 11 participants reported a symptom, which suggests a high false alarm rate for subjective symptom identification.

To observe the relationship between altitude and reported symptoms, while accounting for the slight variability between participants during the gradual incline, the symptom reports were binned by altitude into the following four bins: 0–10k', 10–15k', 15–20k', and 20–25k'. The frequency of the first symptom reported to their corresponding altitude is illustrated in Figure 7B. Though the first symptom was most frequently reported at altitudes from 10–15k', the distribution across altitude bins was relatively even. This even distribution might suggest high inter-individual variability and generally low sensitivity, as a majority of the participants did not recognize their impairment due to hypoxia until well into the exposure (see Section “4” for additional considerations).

We also identified participants' SpO_2_ levels at the time of their first symptom report. Figure 7C shows the frequency of the first reported symptom relative to the SpO_2_ level bin (in percentage: 100–95, 94–90, 89–80, 79–70, and 69–60). The first bin is from 100 to 95, as ≥ 95% is considered normal in healthy adults (Chuiko et al., 2020; Bhutta et al., 2022). No one reported their first symptom at <60%. The average SpO_2_ when the first symptom was reported was 84% (*SD* = 12.13), with the maximum being 100% and the minimum being 61%.

## 4 Discussion

By assessing changes in physiology, cognitive performance, symptom reporting, and neural activity during a normobaric hypoxia exposure, we extended a previous body of hypoxia research using an exposure profile closely related to current protocols for military aviation training. The US Navy is currently transitioning from the Reduced Oxygen Breathing Device (ROBD-2) to the ODHT and newer FBAT device for hypoxia familiarization training. The current study represents one of the first studies to use this new generation of device to assess hypoxia effects on a number of aircrew relevant measures. Our findings help clarify the timing and specificity of hypoxia effects induced by the ODHT on physiology and performance. In particular, we show that symptom recognition was delayed relative to physiological measures and provide further support for the utility of EEG in assessing impacts of hypoxia on brain function.

Physiological measures including HR and SpO_2_ responded within 2–3 min to the start of hypoxia exposure and continued to change roughly linearly as altitude increased. Increased HR and decreased SpO_2_ are well-established responses to hypoxia exposure, known as the hypoxic ventilatory response (e.g., Petrassi et al., 2012). The responses shown for the specific altitude schedule produced with the ODHT, however, are novel and may provide a frame of reference for future ODHT exposures.

A cognitive assessment battery designed for hypoxia exposures, the HAT (SoarTech Inc., 2020), was chosen as the primary performance task for this study. We found that HAT performance decrements due to hypoxia were pronounced at the more extreme compared with less extreme altitudes and were task-specific. Performance on two of the four tasks in the assessment, Level 1 (with subtasks including rule operations, colored addition problems, and fine motor control) and Level 2 (Stroop) was not sensitive to hypoxia, suggesting these tasks may have been too easy and participants experienced a ceiling effect. However, performance on the remaining two tasks (Advanced Stroop and Tracking) was adversely affected at higher altitudes. Performance was similar for these tasks in the later rounds compared with earlier rounds under normoxia, suggesting that the differences were due to hypoxia and not cognitive fatigue, boredom, or other time factors. The Advanced Stroop and Tracking tasks both include a visual search component, while the Advanced Stroop task also relies on inhibitory control and the tracking task relies on working memory. The results suggest that these constructs, when combined with the visual and attentional demands of visual search, were particularly vulnerable to the hypoxia exposure. Other studies have also found adverse impacts of hypoxia on working memory (Malle et al., 2013; Bouak et al., 2018) and Word-Color Stroop performance (Asmaro et al., 2013; Ochi et al., 2018; Post et al., 2023). Future research could investigate further the impact that hypoxia has on higher level cognitive abilities, such as executive function, by utilizing an ERP paradigm that indexes those processes specifically. However, that work would require an active ERP paradigm, which is in contrast to the passively elicited MMN/P3a that we have implemented here with an eye toward application in operational environments.

Symptoms due to hypoxia were reported by participants in a delayed manner following hypoxia exposure and varied widely. This highlights the shortcomings of relying on self-report alone for hypoxia training and detection of hypoxia during in-flight emergencies. This may be due to the distraction of completing the HAT task precluding thorough symptom reporting in real-time, which the effects of hypoxia likely made more difficult. It is worth noting that while the HAT distracts from identifying symptoms, the mental workload of flying an aircraft would similarly pose a concern for distractions as well. A study by Deussing et al. (2011) highlights this shortcoming with participants during a flight simulation task, where only 20% reported experiencing hypoxia symptoms. Given the relative novelty of the ODHT and its role in simulating a hypoxia exposure that may be encountered in tactical aviation, the high number of common hypoxia symptoms also reported in this study attest to the ODHT's ecological validity. A comparison of symptoms reported here to those reported in PEs or other modes of hypoxia training may be worthwhile to inform future efforts using the ODHT.

Neural impacts of hypoxia were evident in the MMN/P3a as a reduction in peak-to-peak amplitude coinciding roughly with the time frame for the first self-reported symptom, around 10 min into the exposure period. While we have shown robust effects of hypoxia on the MMN/P3a complex previously (Seech et al., 2020; Blacker and McHail, 2021), the current study used a novel device (ODHT) and more gradual altitude exposure schedule rather than the immediate and prolonged exposure to 17.5 k' and 20k' previously used. As such, the effects of hypoxia were more modest with the current experimental manipulation, which is not surprising. Incorporating a normoxia control in the current study revealed a time effect with decreasing MMN/P3a amplitude which is likely due to habituation to the auditory stimuli. Habituation to auditory deviant stimuli has been well established (Sörqvist et al., 2012; Littlefair et al., 2022), but the time course of the MMN/P3a is relatively underreported in the literature. Our findings help fill this gap and also support including a normoxia control in future experiments that examine the time course of hypoxia effects to help rule out the contribution of habituation to changes in the MMN/P3a.

As prior work has shown that the MMN/P3a complex is a robust neural marker of hypoxia, real-time detection of changes in the MMN/P3a may be valuable as a monitoring tool during aviation hypoxia training, research, and during flight (if reliable in-cockpit EEG devices become available in the future). Exploratory analyses of the current dataset used a moving window approach to examine variability of the MMN/P3a over time to determine if changes in MMN/P3a amplitude could reliably detect impairment due to hypoxia for an individual participant within a single visit. However, this method proved too noisy and prone to false reports, and results were not reported here. While changes in MMN/P3a amplitude alone were unsuccessful in reliably detecting hypoxia on the individual level, it is possible that changes in other features of the MMN/P3a waveform might be detected using machine learning tools that we plan to employ in the future on this data set. An effective classifier of an impaired state due to hypoxia, whether using the MMN/P3a alone or integrated input from other EEG markers and physiological signals, would be highly beneficial for future hypoxia monitoring devices.

The current study did not address an open question that remains in this area of research, which is the mechanism by which acute hypoxia influences the MMN/P3a signal. The MMN/P3a are known to have two distinct neural generators, namely a bilateral supratemporal component and a frontal component (Naatanen et al., 1978; Giard et al., 1990; Baldeweg et al., 1999). Presently, the literature regarding localization of a neurovascular response during acute hypoxia is inconsistent. Specifically, it has been found that cerebral blood flow (CBF) increases in evolutionary older regions of the brain compared to evolutionary younger areas (Binks et al., 2008), whereas it has also been found that CBF increases in sensorimotor and prefrontal cortices (Pagani et al., 2011). Some studies have also found the hypothalamus to have the largest vascular response (Buck et al., 1998), while other studies demonstrated support for a more unitary vascular response (Dyer et al., 2008; Harris et al., 2013). Inconsistencies in the literature may be due to the level of hypoxia exposure (acute vs. mild; e.g., Harris et al., 2013). Future work using methods suited for localization of the vascular response like functional magnetic resonance imaging and transcranial doppler should be used to investigate this issue further.

One of the limitations of the current study, as well as the majority of the extant literature, is studying specific neurocognitive measures in isolation. It remains unclear how sensory degradation (e.g., Seech et al., 2020), impaired attention (e.g., Blacker and McHail, 2021), and decrements in executive function (e.g., Malle et al., 2013) interact with one another during acute hypoxia exposure. For example, is there a downstream effect from sensory processing changes to attention and ultimately to executive function? Are some individuals more affected in specific sensory modalities (e.g., visual) and other individuals more widely affected in many modalities? These questions are ripe for future work to improve the potential of in-flight physiological monitoring. A second limitation here, which is also common in the literature, is the lack of brain-behavior relationships between our EEG measures and our cognitive performance tasks. Here, we chose auditory ERPs based on established prior findings, but we ultimately chose cognitive tasks that were visual in nature to deconflict the two modalities. We also span the spectrum of preconscious measures with our ERPs to higher-order executive function with tasks like the Stroop. While we could investigate executive function specifically with both cognitive tasks and ERPs that underlie those constructs to better understand human performance during hypoxia, an active paradigm like that would not advance the ultimate goal of developing in-flight monitoring capabilities.

Our findings identified selective impacts of hypoxia on cognitive performance as well as pre-attentive auditory processing and associated neural activity. Our results help advance hypoxia research closer to the current aviation survival training environment and provide a foundation for future hypoxia research using the ODHT.

## Data availability statement

The raw data supporting the conclusions of this article will be made available by the authors, without undue reservation.

## Ethics statement

The studies involving humans were approved by the Naval Medical Research Unit-Dayton Institutional Review Board. The studies were conducted in accordance with the local legislation and institutional requirements. The participants provided their written informed consent to participate in this study.

## Author contributions

CB: Formal analysis, Project administration, Writing – original draft, Writing – review & editing, Data curation. DM: Formal analysis, Writing – original draft, Writing – review & editing, Conceptualization, Methodology, Visualization. KB: Conceptualization, Formal analysis, Methodology, Visualization, Writing – original draft, Writing – review & editing, Funding acquisition, Project administration, Resources, Supervision.
