# Supplementary material for: The time course of hypoxia effects using an aviation survival trainer
Source: Front Cognit. 2024 Apr 10;3:1375919. doi: 10.3389/fcogn.2024.1375919 (PMC13281091; doi:10.3389/fcogn.2024.1375919)
Supplement: Supplementary file 1 [file Data_Sheet_1.docx]

Supplementary Material

# Supplementary Figures


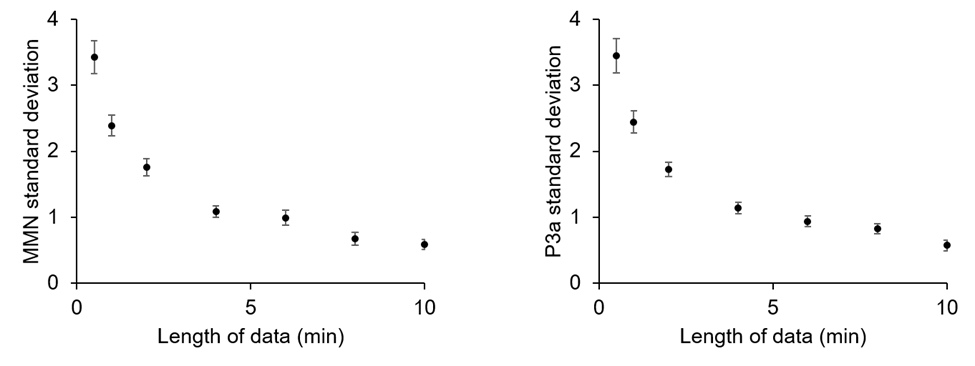


**Supplemental Figure 1.** Standard deviation for MMN/P3a amplitude compared with length of data. Determining an appropriate window size involves a tradeoff between maximum granularity (smaller window) or maximum reliability (larger window). If a smaller amount of data is sufficient, this may also mean a shorter amount of time needed to detect impairment. Data from normoxia visits (~30 min total) were divided into bins from 0.5 min to 10 min in duration. For MMN amplitude (left panel) and P3a amplitude (right panel), the plots show average standard deviations for each data length across participants (2 participants excluded for noise, n=30) with error bars showing SEM. Values converged over a tighter range with increasing length of data, with diminishing returns after 4 min of data. While differences continue to decrease past 6 min for both the MMN and P3a, 6 min was chosen as the best window length for this analysis to maximize granularity and reliability in the data.
